# Alterations of Photosynthetic and Oxidative Processes Influenced by the Presence of Different Zinc and Cadmium Concentrations in Maize Seedlings: Transition from Essential to Toxic Functions

**DOI:** 10.3390/plants13081150

**Published:** 2024-04-20

**Authors:** Ildikó Jócsák, Ferenc Csima, Katalin Somfalvi-Tóth

**Affiliations:** Institute of Agronomy, Hungarian University of Agriculture and Life Sciences, 7400 Kaposvár, Hungary; csima2001@gmail.com (F.C.); somfalvi-toth.katalin@uni-mate.hu (K.S.-T.)

**Keywords:** essential–nonessential comparison, zinc deficiency and toxicity, cadmium toxicity, maize delayed-fluorescence (DF), ultra-weak bioluminescence (UWLE)

## Abstract

Background: The study examined the impact of varying the concentrations of zinc (Zn) on plant responses, particularly on photosynthetic and oxidative metabolic processes. This investigation aimed to distinguish between the beneficial and harmful effects of Zn on plants, highlighting significant nutrient supply concerns. Methods: The investigation methods were centered around non-invasive methods, such as biophoton emission (delayed fluorescence—DF, ultra-weak bioluminescence—UWLE), fluorescence induction (F_v_/F_m_) measurements, chlorophyll content estimation (SPAD) and vegetation index (NDVI) determination. Furthermore, the analytical determination of lipid oxidation (MDA level) and antioxidant capacity (FRAP) as well as gene expression studies of the antioxidative enzymes glutathione reductase (GR), glutathione S-transferase (GST) and lipoxygenase (LOX) for essential Zn and nonessential cadmium (Cd) were also carried out in order to clarify toxic symptoms through different Zn investigation approaches. Results: It was possible to identify a metabolic enhancement from 1000 µM; however, stress symptoms from the 2000 µM Zn treatment were noted for both the investigated photosynthetic and oxidative processes. The outcomes of this research contribute to the improvement of Zn mineral-supplementation technology, which is essential for maize growth, and the optimization of agricultural practices.

## 1. Introduction

Maize (*Zea mays* L.) is an important crop of *Poaceae* family that is cultivated worldwide in temperate and tropical locations. Maize is mostly utilized for animal feed, silage, and fine chalk manufacturing [1].

Nutrient supply is an important issue in which Zn, as a heavy metal and essential micronutrient, plays a role of particular importance in maize production. Heavy metals, depending on their nature, can swiftly enter plants from soil and alter plant metabolism, determining maize development. This element group (including copper, manganese, lead, cadmium, nickel, cobalt, iron, zinc, chromium, arsenic, and silver) comprises metals and metalloids with atomic densities > 4 gcm^−3^. The presence of heavy metals affects several metabolic pathways. Excessive heavy metal concentrations in soil can reduce soil enzyme activity [2,3]. Greger (2004) classifies uptake as shoot accumulators (Ag, Cr, Pb, Al, Va, Sn), root accumulators (Cd, Co, Cu, Fe, Mo), and uniform distributors (Ni, Mn, Zn) [4]. Due to their microelement nature, essential metals, such as, e.g., Zn that is an important nutrient of maize, also benefit plant function. Heavy metals induce oxidative stress, which hinders plant survival. Redox-active metals produce hydroxyl radicals through Haber–Weiss reactions [5], whereas Zn and Cd cannot [6].

Zinc (Zn) is an essential heavy metal for maize that is used to compensate for soil Zn deficiencies, which can reduce yield and quality. Zn deficiency decreases protein synthesis [7] due to RNA degradation [8] and RNA polymerase activity, ribosome deformation, and ribosome number, limiting plant health and viability, resulting in reduced growth, yields (or crop failure), and crop quality [9]. Zn-deficient plants have defective protein, carbohydrate, and auxin metabolism [10]. Zn is the only metal needed in all six classes of enzymes (oxidoreductases, transferases, hydrolases, lyases, isomerases, and ligases). It is an enzyme-former and activator, integrating into Ribulose-1,5-bisphosphate carboxylase/oxygenase (RuBisCO); the antioxidant system is also affected by Zn supply, including superoxide dismutase (SOD), ascorbate peroxidase (APX), and glutathione reductase (GR) enzymes, ascorbic acid, and glutathione (GSH). Protein, chromatin and DNA/RNA metabolism and gene expression processes require Zn; therefore, it is crucial to protein synthesis, stability, and the function of genetic material [11,12].

Excess Zn availability, however, is harmful for maize, by reducing plant weight, length, and development due to cell division inhibition [12,13,14]. High Zn concentrations diminish chlorophyll A, B, and A/B ratio and hinder the efficiency of photosystem I (PSI) and photosystem II (PSII) [15]. Zn stress generates reactive oxygen species (ROS) and impairs metabolic activities like the antioxidant defense system and photosynthetic electron transport [16]. Lipid peroxidation and membrane permeability also increase with Zn-induced oxidative stress [17]. Furthermore, Zn enhances lipoxygenase (LOX) activity during oxidative stress, which may activate plasma membrane NADPH oxidase and increase superoxide anion generation [18]. The activities of the antioxidant systems such SOD, APX, and GR also affect Zn content [19].

Nonessential heavy metals, such as Cd, which reach plants through divalent ion channels of essential elements like calcium, cause unfavorable metabolic processes [20].

Cadmium is mostly produced as a byproduct of Zn, Cu, and Pb extraction and refining. The use of Cd-containing products and fuels and the lack of recycling contribute to soil contamination, mostly through atmospheric deposition near primary sources of air pollution (mining and refining, incinerators, fossil fuel burning) and technological waste dumps [20,21,22]. Cd is one of the most hazardous heavy metals in the environment because it is extensively translocated and poisonous even at low concentrations [23,24]. Cd accumulates mostly in the root, but also translocates into shoots. Cd hinders maize seedling growth and germination [25,26]. Cd stress causes ROS production, including superoxide anion radicals (O_2_^−^) from molecular oxygen through single-electron reduction [27,28]. Multiple studies have revealed that Cd and other heavy metal stressors cause maize lipid peroxidation [20,25,29,30,31,32,33].

Plant stress research relies on nondestructive or noninvasive technologies to test the same plant multiple times and characterize the basic physiological processes that cause stress phenomena without damaging the plant tissue.

In vivo and noninvasive assays are crucial for plant stress detection, because plants can be investigated multiple times during development to observe changes without harm. Biophoton emission measurement has grown in popularity during the past years [34,35,36,37,38], as a new noninvasive visualization tool for plant stress is biophoton emission measuring via photosynthetic and oxidative biochemical activities. This method enables the study of the state and function of PSII and oxidative processes via delayed fluorescence (DF) and ultra-weak bioluminescence (UWLE), respectively.

When green plants are placed in the dark, the excitation energy ceases, and the cessation of water splitting leaves the PSII without an electron donor and in an electron-deficient state. Therefore, electrons from the electron transport chain return to the reaction center, excite chlorophyll molecules, causing photon emission [39,40]. Photoluminescence from plant cells is related to the oxidative metabolism during which UWLE signals are produced compared to DF and produced by lipid peroxidation processes increasing UWLE [40]. Former studies have shown that with biophoton emission detection, it is possible to determine the plant stress reactions caused by Cd, herbicides and insect infestation [40,41,42].

However, this method delivers rapid information without being selective for metabolic processes [40]; therefore, it is advised to assess the underlying metabolic processes of the overall photon emission signals. Plant oxidative stress can be assessed by several stress markers, such as malondialdehyde (MDA) or lipoxygenase (LOX) levels to quantify the rate of lipid oxidation. Polyunsaturated fatty acids react with oxygen via lipoxygenase (LOX) enzymes resulting in the formation of oxylipins [43,44,45,46]. Several LOX isoforms have evolved [47] of which the soluble-cytoplasmic fraction has the highest activity [48]. Oxylipin products are essential to plant metabolism, including growth, stress responses, senescence, and cell-to-cell signaling [49]. LOX2 gene production promotes the generation of abiotic and biotic stress-defense mechanisms [50].

To better comprehend the mechanisms of non-enzymatic antioxidants, we examined the total ferric reducing antioxidant power (FRAP) [51]; additionally, the expression of the genes of antioxidant enzymes (glutathione S-transferases—GSTs; glutathione reductase—GR) was determined, in which GSTs detoxify xenobiotics and endogenous substances, converting them to less poisonous forms by linking them to glutathione [52,53,54] and GR catalyzes the reduction in oxidized glutathione (GSSG) to its reduced form glutathione (GSH) using NADPH as the reducing cofactor, and thereby maintaining a constant GSH level in the system [55,56].

The current investigation targeted the important nutrient supply-related issue of heavy metal microelements: the distinction between the essential and toxic effects of Zn, focusing on how different concentrations (100, 500, 1000, 2000 µM) affect the maize plant’s metabolic responses, and specifically on the effects on photosynthetic and oxidative processes. Additionally, due to the similar geochemical and environmental characteristics of Cd and Zn [22,23,57,58,59], although it is a nonessential heavy metal, Cd was also included in the study (100, 500 µM) to accurately characterize the pure toxic symptoms. The experimental hypothesis was defined as follows: low (100 µM) Zn treatment shows results indicative of deficiency symptoms, i.e., lower initial DF values and increased and higher (2000 µM) concentrations already cause the appearance of toxic symptoms. The two cadmium treatments (100, 500 µM) were included in the study to monitor, control and confirm the toxic symptoms, as this metal only induces metabolically damaging processes due to its nonessential nature. The exploration of the photosynthetic and oxidative metabolic processes was conducted through non-invasive and analytical investigations, also through gene expression studies, in the hope that the outcomes contribute to advances in nutrient supplementation technology for Zn, an essential component of maize growth, in order to enhance agricultural practices.

## 2. Results

### 2.1. Results of the Principal Component Analysis

Statistical techniques were selected at the beginning of the studies to isolate the effects of the treatments used in the experiment before analyzing each test parameter individually.

**Figure 1:**
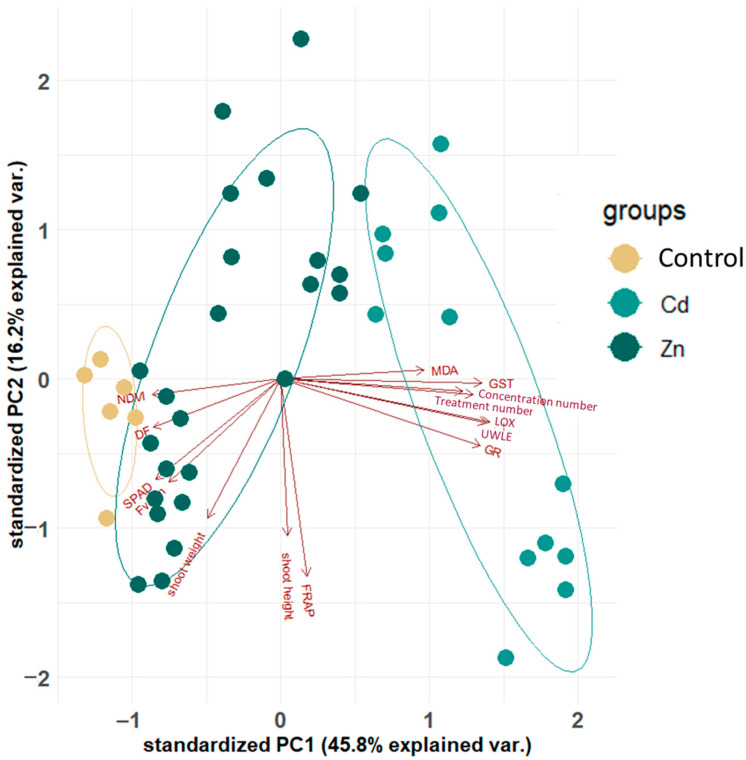
Visualization of the PCA results. Clustering the PCA results by the treatment (colors and circles) as the most significant factor among the studied variables. The significance (length of the red rays) and the correlations of the studied parameters (the arrows’ position to each other) show that the concentration and treatment (control, Zn, Cd) vary together with GS T, UWLE, LOX, MDA and GR along the first principal component (PC1), while shoot weight and height, and FRAP have a strong relationship.

Principal component analysis (PCA) examines the significance of each parameter and its relationship to the others. The role of these parameters in the coordinate system represented by the first and second principal components is illustrated in Figure 1. The first principal component increases with increasing concentration, treatment, GST, UWLE, LOX and MDA scores. GR has a lower weighting on the processes. This suggests that these seven criteria vary together. If one increases, then the remaining ones tend to increase as well. On the other hand, the values of the NDVI, DF, SPAD, and F_v_/F_m_ parameters increased with the decrease in concentration and treatment. The second principal component shows that the shoot weight, shoot height, and FRAP react together; with the increase in shoot weight and shoot height, the values of FRAP increase as well. The clustering of the values by the treatment (control, Cd, Zn), as one of the most significant factors on the variance, can be seen in Figure 1.

**Figure 2:**
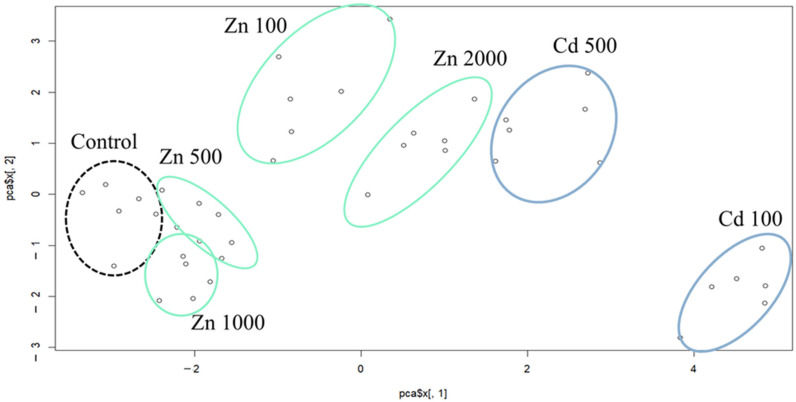
The values of the first and second components grouped by the mutual effect of concentration and treatment.

The values of the first and second components are grouped by the mutual effect of concentration and treatment (Figure 2). The location of the control group can be seen as a starting point with an average shoot weight and shoot height and with the highest values of DF and NDVI. The most similar groups to the control group were Zn 500 and Zn 1000. The location of the circle of Zn 100 shows that the shoot weight, shoot height, and FRAP values were the lowest in this group. The Zn 2000 had a similar effect to the Zn 100 but with a lower degree of decrease. The Cd 100 group is distinct from all other groups. The PC1 values were the highest, and the PC2 values were the lowest, which means that the values of MDA, GST, UWLE, LOX and GR were the highest together with the shoot weight, shoot height and FRAP values as well. All in all, the values of PC1 varied significantly among the different treatment groups (control, Zn, Cd), while the concentrations had a more complex effect on the results.

### 2.2. Element Accumulation, Shoot Height and Shoot Weight

**Figure 3:**
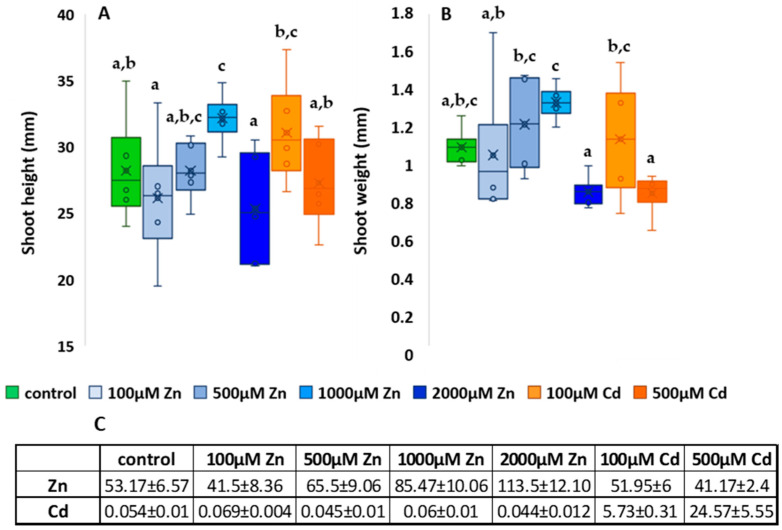
Shoot height results (**A**), and shoot weight results (**B**) and element accumulation (mg/kg dry weight) (**C**). The results are presented as an average of the values of each treatments (n = 5) ± standard deviations (SD). Different lowercase letters indicate significant difference (*p* < 0.05).

Plant height, weight and element accumulation are shown in (Figure 3A,C). The element accumulation data indicate that both zinc (Zn) and cadmium (Cd) were found in the aboveground sections of the plant. and the rate of accumulation increased with increasing metal concentration (Figure 3C). The Zn accumulation was proportional to the concentrations used and the 100, 500, 1000 µM Zn treatments resulted in an increase (500 µM Zn: 23%; 1000 µM Zn: 60.7%; 2000 µM Zn: 113%); only the 100 µM Zn treatment resulted in a decrease in maize leaf Zn content compared to the control. The Zn content of the Cd-treated plants did not differ from the Zn content of the control plants. Cd accumulation was also proportional to the concentrations applied, and compared to the control, 100 µM Cd treatment increased the Cd content in maize leaves by one order of magnitude, while 500 µM Cd treatment increased it by two orders of magnitude. The Cd content of the Zn-treated plants did not differ from the Cd content of the control plants.

The mean plant height of the control group was 28.18 ± 1.88 cm, which was the same as the 500 µM Zn-treated group (28.18 ± 1.05 cm). The average height of the 100 µM Zn-treated group was 26.12 ± 2.23 cm which was 7.3% lower than the control and could be a consequence of nutrient deficiency.

The 2000 µM Zn-treated group was the lowest with an average height of 25.32 ± 1.97 cm and the 1000 µM Zn-treated group was the highest with a height of 32.12 ± 0.9 cm, a significant difference from the control. Of the Cd treatments, the lower dose induced a significant increase (31.04 ± 1.85) and the higher dose-treated group was 3.2% lower than the control. The results show that the 100 µM and 2000 µM Zn treatments resulted in a statistically verifiable reduction compared to the control, and the higher 500 µM Cd treatment (Figure 3A).

As for shoot weight, the group with the highest mean value was 1000 µM Zn with 1.29 ± 0.04, that is 22.86% higher than that of the control. However, the mean of the treatment with the highest doses of Zn (2000 µM) and Cd (500 µM) were both 23.2% lower than the control and the difference was also confirmed by the statistical analysis. All the other treatments did not result in statistically proven differences (Figure 3B).

### 2.3. Photosynthesis-Related Studies

**Figure 4:**
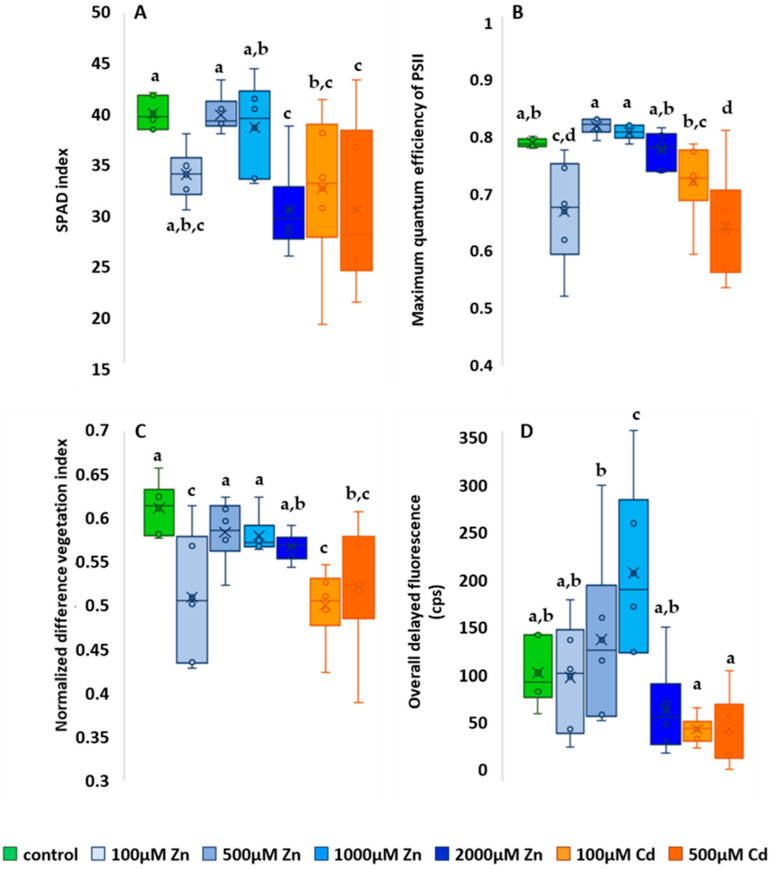
Effect of zinc and cadmium treatments on chlorophyll content (**A**), fluorescence induction (**B**), normalized vegetation index (**C**) and delayed fluorescence (**D**). The results are presented as an average of the values of each treatments (n = 5) ± standard deviations (SD). Different lowercase letters indicate significant difference (*p* < 0.05).

The results of the chlorophyll content estimation are shown in Figure 4A. The control value was 40.08 ± 0.9, compared to which all treatments resulted in a lower average. The plants treated with the two highest concentrations (500 µM Cd and 2000 µM Zn) had the lowest average chlorophyll content, which was 30.5 in both cases. This was 24.1% lower than the control average and clearly indicated heavy metal stress. The highest Zn concentration treatment and the two Cd treatments resulted in significantly lower chlorophyll content than the control (*p* = 0.002). Also, the 100 µM Zn-treated plants had a lower mean (34 ± 1.27), 15.2% lower than the control, presumably due to nutrient deficiency.

The results of the F_v_/F_m_ measurements are shown in Figure 4B. Fluorescence induction measurement summarized results showed higher F_v_/F_m_ values than the control (0.78 ± 0.007), including for 500 µM Zn (0.8 ± 0.014) and 1000 µM Zn (0.8 ± 0.006)-treated plants. This shows that Zn supplementation with this amount had a positive effect on photosynthetic activity. The lowest mean F_v_/F_m_ values were measured for the 500 µM Cd treatment (0.6 ± 0.09), which was 23.1% lower than the control. Zn applied at the lowest concentration (100 µM) showed a significantly lower mean of 0.66 ± 0.09, 15.4% lower than the control (*p* < 0.001). The smallest difference from the control was found for the highest Zn treatment (2000 µM) (0.77 ± 0.02), showing a difference of only 1.3%. Accordingly, our fluorescence induction results suggest that only Cd treatment induced a toxic effect, and the results for plants treated with the lowest Zn concentration (100 µM) are probably due to nutrient deficiency, as well as chlorophyll content.

For NDVI measurements (Figure 4C), all treatments also resulted in a lower mean than the control (0.61 ± 0.015), and again a mean indicative of nutrient deficiency was observed for the lowest Zn treatment (0.51 ± 0.04), this time also significantly (*p* = 0.001), by 16.4%, lower than the control. For higher Zn treatments, a slight downward trend in the means was observed: 500 µM = 0.584 ± 0.018; 1000 µM = 0.58 ± 0.01; 2000 µM = 0.57 ± 0.008. The lowest NDVI values were observed for plants treated with 100 µM Cd (0.5 ± 0.02), 18.1% lower than the control.

The overall results of the ten-minute DF are shown in Figure 4D. For the overall DF, the mean of the control was 114.79 ± 17.1. Again, the mean was slightly lower for the lowest dose of Zn treatment (110.36 ± 28.9). Two Zn treatments resulted in elevated mean DF values. Compared to the control, the 500 µM (149.7 ± 45.2) nutrient treatment had a 30.4% higher photon emission in the first minute compared to the control, and the 1000 µM (219.9 ± 45.1) treatment had a 91.6% higher photon emission in the first 10 min, and was the only one that showed a significant difference from the control (*p* < 0.001). However, plants treated with the highest Zn dose (2000 µM) had reduced DF values, averaging 75.94 ± 23.6, which was 33.8% lower than the control. This clearly shows that while two Zn treatments had a positive effect on photosynthetic activity, the highest treatment applied had a negative effect on photosynthesis and plant physiology.

Similar to the latter, nonessential Cd treatments also resulted in reduced DF values. The mean values were 51.7% lower for the 100 µM Cd treatment (55.5 ± 7.98) and 51% lower for the 500 µM Cd treatment (56.2 ± 18) compared to the control.

**Figure 5:**
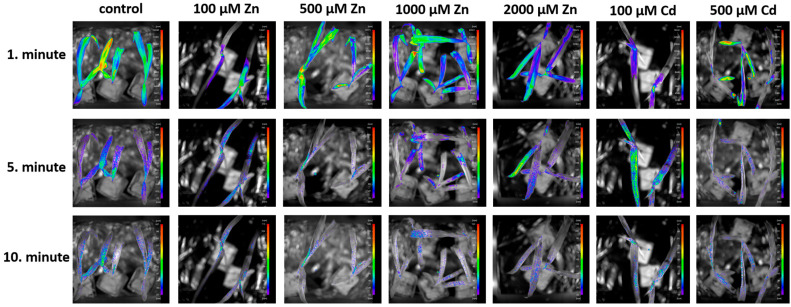
Changes in delayed fluorescence on the 1st, 5th and 10th minute of the measurement. The color intensity represents the biophoton signal strength measured by the equipment and translated into a color intensity scale using IndiGoTM 2.0.5.0 software.

The images (Figure 5) show the changes in DF on the 1st, 5th and 10th minute of the measurement in order to illustrate the visually observable changes in DF values. The color intensity represents the biophoton signal strength measured by the equipment and translated into a color intensity scale using IndiGoTM 2.0.5.0 software. In all cases, the decrease in DF values can be deduced from the outputs of the visualizations (Figure 5), and there are differences between the effects of each treatment and the control signal intensities for the maize plants. In each image, the highest signal intensity emitted by the plants is indicated by the color red and the lowest by the color purple, and the maximum values for each pixel-color intensity are quantified on the scale assigned to the images.

**Figure 6:**
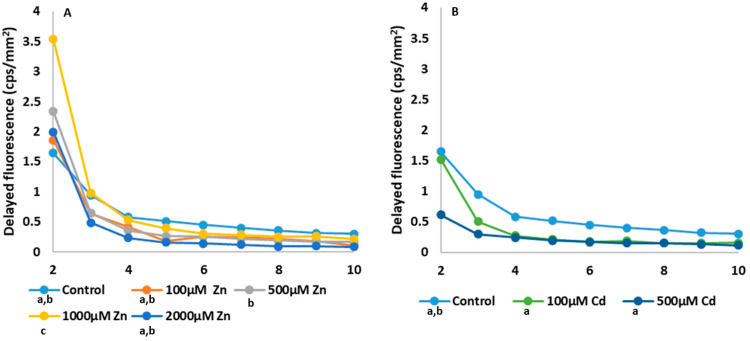
Time course of delayed fluorescence of Zn (**A**) and Cd treatments (**B**), starting from the 2nd minute of the measurement since the values of the first minute (Figure 4D) are expressed in counts per second (cps) values per mm^2^. The changes in photon emission values in the first minute are so high that they would mask the dynamics of the decrease in DF. The values presented in these diagrams are the averages of five independent measurements.

The following figures show the changes in the DF values and their dynamics over time (Figure 5 and Figure 6A,B). In addition to the magnitude of the initial fluorescence values, their decay time and decay time provide information about the state of the photosynthetic system. The figure shows the separation of the initial values and the differences in decay. Although the Zn-treated plants showed higher values than the control in the first minute, the decay was faster in plants treated with 100 µM and 2000 µM Zn, which implies that the overall DF is also lower than the control. Cd-treated plants also had a lower photon emission than the control in the first minute, followed by a faster decay, resulting in the lowest overall DF.

### 2.4. Oxidative Metabolism-Related Studies

**Figure 7:**
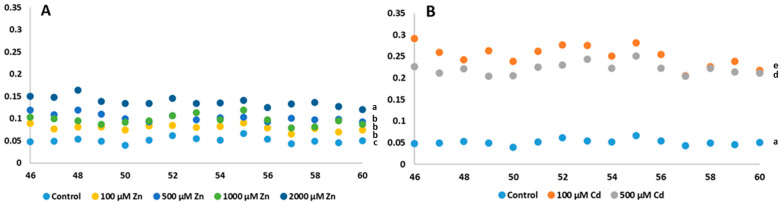
Time course of the changes in ultra-weak bioluminescence of Zn (**A**) and Cd treatments (**B**) from the 46th to the 60th min of the measurements expressed in counts per second (cps) values per mm^2^ (cps/mm^2^). The values presented in these diagrams are the averages of five independent measurements. Different lowercase letters indicate significant difference (*p* < 0.05).

The UWLE range of photon emission, which is characteristic of oxidative metabolic processes, was recorded after the DF phenomenon had decayed, at between 30 and 60 min of the measurements (Figure 7A,B).

**Figure 8:**
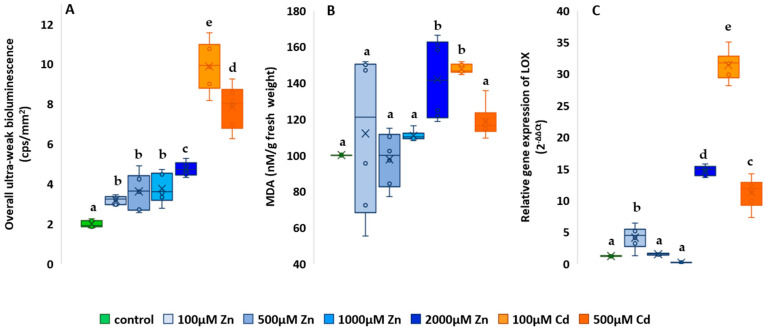
Effect of zinc and cadmium treatments on ultra-weak bioluminescence (**A**), lipid oxidation (**B**) and LOX gene expression (**C**). The results are presented as an average of the values of each treatment (n = 5) ± standard deviation (SD). Different lowercase letters indicate significant difference (*p* < 0.05).

In all cases, the UWLE values of the heavy metal-treated plants were higher than the mean of the control (1.98 ± 0.09), shown in Figure 7A. An upward trend in photon emission was observed throughout for the Zn treatments. The lowest increase was observed for the lowest dose treatment (60.6%) and the highest for the highest dose (138.89%). Much higher values were observed for Cd-treated plants. The 100 µM treatment showed an average of 9.88 ± 0.68 and the 500 µM treatment an average of 7.84 ± 0.54, a significant increase of 399.49% and 295.45%, respectively (*p* < 0.001). The differences can also be seen in Figure 8A, where the plants treated with Cd show the highest photon emission after 30 min (Figure 7B). From these results, it can be seen that all treatments increased oxidative metabolism in plants, especially Cd treatments.

MDA results are shown as a percentage of the control (Figure 8B). The 500 µM Zn treatment was the only one to show a lower value than the control (97.66 ± 7.32). The highest difference among the Zn treatments was shown for the 2000 µM treatment with 141.7 ± 11.15 (*p* < 0.001). The two Cd treatments used also showed an increase in lipid oxidation compared to the control. Similar to the UWLE results, a greater increase was observed for the 100 µM treatment (147.55 ± 1.33) than for the highest treatment (118.48 ± 4.49).

The results of the LOX gene expression assays are shown in Figure 8C. The relative gene expression of the control was 1.009 ± 0.04. Only the 1000 µM Zn treatment resulted in lower values. Similar to the UWLE and MDA results, the lowest Zn treatment gave higher values than the control, LOX assays also showed significantly higher relative gene expression at 3.94 ± 0.9 (*p* < 0.001). In addition, the results also clearly showed the toxic effect, with both the highest Zn and the two Cd treatments showing a significantly higher relative gene expression than the control.

### 2.5. Studies on the Antioxidant System

**Figure 9:**
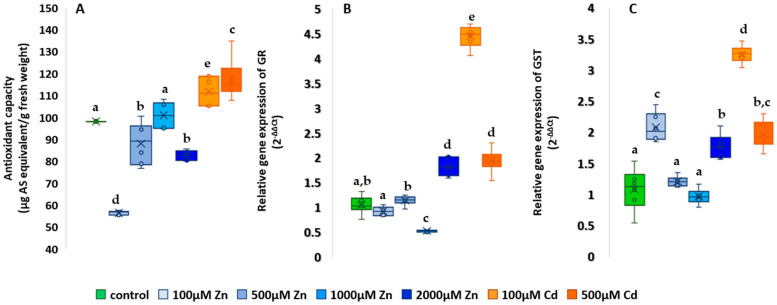
Effect of zinc and cadmium treatments on antioxidant capacity (**A**), GR (**B**) and GST gene expression (**C**). The results are presented as an average of the values of each treatments (n = 5) ± standard deviations (SD). Different lowercase letters indicate significant difference (*p* < 0.05).

The results of FRAP measurements are presented as a percentage of the control (Figure 9A). Of the Zn treatments, only 1000 µM had a higher antioxidant capacity (102.7 ± 3.08); the other Zn nutrient concentrations showed significantly lower values compared to the control (*p* < 0.001). The lowest non-enzymatic antioxidant amount was found for the lowest Zn treatment, with an average of 59.67 ± 0.465. The two highest values were found in the two Cd-treated groups, with a 13% increase in antioxidant capacity at 100 µM and an 18.5% increase at 500 µM, which is statistically significant compared to the control (*p* < 0.001).

The results of the GR gene expression studies are shown in Figure 9B. In GR assays, the relative gene expression of the control was 1.04 ± 0.09. The 1000 µM Zn treatment resulted in the lowest relative gene expression in both antioxidant system gene expression assays, 50% lower than the control for GR and 10.1% lower for GST. For GR, the toxic effect of heavy metals was well demonstrated, as the 2000 µM Zn and the two Cd treatments resulted in the three largest increases compared to the control. The highest Zn treatment showed 73% and the highest Cd treatment 84.6% higher relative gene expression, both significantly higher than the control (*p* < 0.001). The 100 µM Cd treatment showed the highest relative gene expression (4.42 ± 0.11).

For GST, the mean of the control was 1.09 ± 0.17, as shown in Figure 9C. Only the 1000 µM Zn treatment showed a lower relative gene expression (0.98 ± 0.06). The 2000 µM Zn nutrient solution showed an increase of 64.2%, the 100 µM Zn 91.3% and the 500 µM Cd treatment 80.73%, all significant compared to the control (*p* < 0.001). Similar to the MDA, UWLE and GR assays, the highest values indicative of oxidative stress are observed with the 100 µM Cd treatment, where the mean relative gene expression increased by 199% compared to the control.

### 2.6. Results of the Correlation Analysis

**Figure 10:**
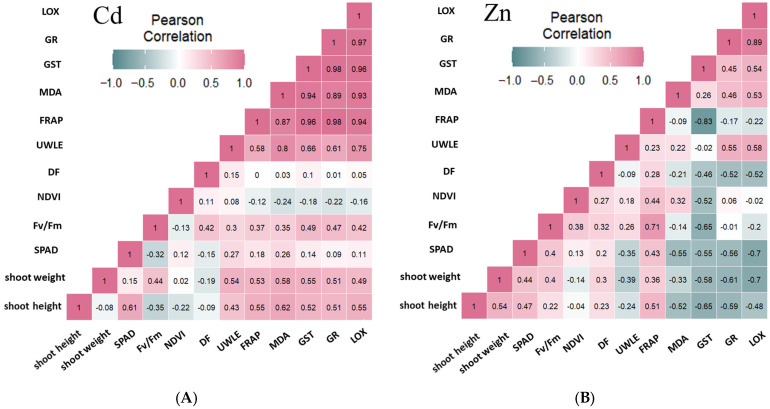
The Pearson correlation coefficients between variables in the case of the presence of cadmium (**A**) and zinc (**B**) separately.

The heat maps illustrate the relationship between the various parameters, as shown by the Pearson correlation coefficients (Figure 10) and summarized in Table 1. The correlation matrices in this section examine the effects of Cd and Zn separately, whereas in the previous section (Figure 1 and Figure 2), they were examined jointly.

The results of the correlation analysis are presented in Figure 10, with the effects of each metal separately (Figure 10A,B). Analysis of the presented parameters and the test results also indicated a correlation between the photosynthetic and oxidative process signals, leading to the assumption of a mathematical relationship between them. The outputs of the Pearson correlation analysis supported this conclusion. The analysis considered the correlation coefficient to be higher than 0.5 in order to include the moderate (0.5–0.599), strong (0.6–0.799) and very strong (0.8–1.00) relationships as well.

With respect to Cd, it can be seen that the strongest positive correlation was between the parameters characterizing oxidative processes, with values above 0.9 in most cases (Figure 10A), and also that the correlation coefficients for shoot height and weight were higher than 0.5 in most cases. In contrast, we found weaker correlation coefficients for Zn Figure 10B), and among the oxidative parameter combinations, LOX-UWLE, GR-UWLE, LOX-MDA, LOX-GST, and LOX–GR showed values higher than 0.5; the strongest positive correlation was found between LOX and GR gene expression changes, and the strongest negative correlation was found in the GST–FRAP interaction. Photosynthetic (NDVI, F_v_/F_m_, SPAD) and growth (shoot mass and height) parameters showed negative relationships higher than 0.5 or close to 0.5 (for DF), which were also observed for SPAD, shoot mass and height, and MDA, GST, GR, and LOX combinations. As for the latter parameters, it must be highlighted that the correlation between the plant parts, like the shoot weight and shoot height, and the oxidative processes, like MDA, GST, GR and LOX, showed positive moderate relationships for Cd, which changed to a negative moderate to strong relationship in the case of Zn.

## 3. Discussion

The experimental objective of this work was to separate the essential and toxic effects of different Zn concentrations, as this metal is essential for maize development but also causes degrading symptoms when present in deficiency [7,8,9,10] or in excess [13,14,15,16,17,18,60].

### 3.1. Element Accumulation

The element accumulation results showed that both Zn [61] and Cd [20] reach the aboveground parts of maize plants. Zn is an evenly distributed type of heavy metal [4], and it was justified earlier that Cd is also translocated upward to the shoot proportionally to the applied concentrations [62]. Therefore, as it was justified in earlier research works for both Zn [63,64] and Cd [20,40,65], since the experimental conditions were standardized, the changes in photosynthetic and oxidative processes reflect the heavy metal treatment-induced physiological changes in maize plants.

### 3.2. Justification of the Investigation Methods

The investigation methods are primarily based on non-invasive techniques of the photosynthetic system: relative chlorophyll content estimation (SPAD), normalized difference vegetation index (NDVI), maximum quantum efficiency of PSII based on chlorophyll fluorescence induction (F_v_/F_m_), and biophoton emission measurement. These non-invasive techniques were complemented with analytical methods: spectrophotometric determination of the total antioxidant capacity (FRAP) and lipid oxidation (MDA) and gene expression assays (GST, GR, LOX) centered around the induced oxidative processes in order to elucidate the underlying mechanisms.

### 3.3. Analysis of the Changes in Photosynthetic Processes

In the first phase of the analysis, the photosynthetic status and processes of the maize plants were investigated. The chlorophyll content estimation previously proved to be successfully applied in treatments with well-defined differences or long-term physiological changes, such as differences in nutrient supply or senescence processes [66], and the present work indicated a significant decrease after 6 days of metal treatments in the case of 2000 µM Zn, also for the 100 and 500 µM Cd concentrations, but the lower Zn concentrations (100, 500 µM) were not distinguishable from the control. For these reasons, we intended to extend the investigation by monitoring the NDVI, chlorophyll fluorescence, and biophoton emission changes that reflect not only the amount of chlorophyll but also provide information about the functioning of the photosynthetic apparatus [40,41,42]. As a result of the NDVI measurements, it was revealed that both Zn and Cd treatments reduced NDVI at lower concentrations, which is in agreement with previous experimental results for Zn [67]; where this parameter was reduced, the development of Zn deficiency in the maize was indicated. However, the same decrease could also be a sign of the toxicity of Cd [68], which is in line with the results of Sridhar et al. [69], who investigated phytoremediation processes by searching for spectral signals under Zn and Cd stress in barley plants that indicated the effects and content of heavy metals in the leaves. Their results, however, failed to distinguish between plants treated with different metals on the basis of the normalized differential vegetative index. However, in the present study, we successfully distinguished the Zn and Cd effects on the basis of the NDVI results.

Comparing the changes in fluorescence induction with the results of the chlorophyll estimation, we conclude that they better characterize the effects of the metals tested on maize. The F_v_/F_m_ parameter of fluorescence induction has been used in a number of plant physiological studies [40,70,71,72], and our results confirm theirs, but it should be added that only in cases where the metal effect is pronounced enough to be toxic, such as in the case of Cd; or in the case of Zn treatments, presumably as a result of deficiency reactions [50,73].

Although the effects of the two different kinds of metals were possible to separate using the SPAD, NDVI, and F_v_/F_m_ values, these parameters did not prove to be sufficiently sensitive to monitor the physiological changes induced by lower Zn concentrations. Therefore, the next step of the investigation targeted the biophoton-emission-related analysis.

DF is only present in photosynthetic tissues [38,40,41,74,75] and has decay durations ranging from milliseconds to minutes [39], indicating the condition of the photosynthetic system. During this phenomenon, under dark conditions, electrons in the photosynthetic electron transport chain return to the reaction center of PSII, resulting in excited chlorophyll molecules, which then return to their ground state, releasing photons in the meantime. Studies have shown that the initial values and the decay kinetics of DF can be used to determine the homeostatic status of plants in vivo and estimate stress levels as well [39,42,76]. In the work of Gerhardt and Bodemer [77], DF decay kinetics were characteristic of a stressed *Chlorella* spp. compared to an unstressed culture. This decay time was similar to that observed by Lukács et al. [42] and Jócsák et al. [76]; furthermore, the stressed plants had shorter decay kinetics compared to the unstressed samples [76]. A similar phenomenon was also observed in the present work, when the higher (2000 µM) Zn and both Cd concentrations (100, 500 µM) resulted in decreased initial DF values, indicating lower photosynthetic activity as a consequence of metal-induced stress that is evident for the nonessential Cd, but according to our results, the 2000 µM Zn treatment also initiated stress symptoms that were possible to identify.

It was the 1000 µM concentration that showed the most noticeable increase in DF, which is a sign of a more active photosynthetic apparatus. This is in accordance with previous findings, when short-term elevated temperatures [74] and the application of metabolically stimulating pesticides [75] resulted in higher initial DF values in healthier plants with better defense mechanisms.

Since DF is a dynamic parameter that not only reveals the chlorophyll content, such as the SPAD index, which indicates the total chlorophyll content including functioning and non–functioning pigments as well, this parameter specifically reflects the characteristics of the actual functioning photosynthetic pigments, as described by Berden-Zrimec et al. [78] in their work on the algal population change dynamics of DF. These stress-induced lower DF dynamics were observed not only in the initial DF values but also later, during the ten-minute measurement, in the faster decay of the DF compared to that of the control in the case of the 1000 µM Zn treatment, and as proposed for the toxic Cd in the case of both the 100 and 500 µM treatments. Dąbrowski et al. (2023) [79] investigated the photosynthetic efficiency of *Lolium perenne* L. seedlings in response to Ni and Cd stress and found that heavy metal stress resulted in a decrease in photosynthetic electron transport efficiency, which is consistent with the results obtained in the present work. There was a decreased initial and faster decay of the DF signal, highlighting the importance of the application of chlorophyll fluorescence data from imaging spectroscopy records during the early seedling development, as suggested by Kalaji and Loboda (2007) [80] and Zivcak et al. (2017) [81]. The parameters of the energy fluxes within PSII were quickly shifted (24 h) especially after the application of cadmium [80].

### 3.4. Analysis of the Changes in Oxidative Metabolism-Related Processes

Ultra-weak bioluminescence (UWLE) is a distinct aspect of the general phenomenon of biophoton emission in plants. During dark adaptation, photosynthetic processes gradually stop, after which it is possible to detect an extremely low intensity of UWLE, providing information about oxidative processes in the chloroplast and mitochondria [39]. The bioluminescence values were previously found to increase during the development of stress states in plants [34,42,82], as shown in the case of Cd treatment, when the bioluminescence values were approximately five times as high as the control and twice as high as the UWLE values of the highest Zn concentration. These results were also confirmed by further oxidative stress-related findings.

LOX gene expression is an indicator of lipid oxidation, as proven and visualized by Prasad et al. [34], who investigated a LOX2 mutant of Arabidopsis that lacks the chlorophyll lipoxygenase enzyme and identified that this enzyme is the primary producer of singlet oxygen (^1^O_2_) and triplet carbonyl (^3^L = O*) groups produced during the oxidization of polyunsaturated fatty acids, leading to UWLE during neutralization processes. The increase in UWLE and LOX gene expression, similarly to MDA values, in the case of 2000 µM Zn and 100 and 500 µM Cd concentrations, were all clear indications of oxidative stress.

Furthermore, the functioning of the antioxidant system was characterized spectrophotometrically by the changes in the non-enzymatic antioxidants via FRAP values and by the gene expression changes in GR and GST, and the changes provided insight into the different aspects of antioxidative processes. FRAP values increased for Zn concentrations of 500, 1000, and 2000 µM, which suggests that Zn treatment has a beneficial effect on the function of the antioxidant system, which was concluded earlier by Feigl et al. [83]. In contrast, a significantly lower FRAP value was only observed for 100 µM Zn, which is presumably a consequence of inhibited enzyme activity, such as oxidoreductases, transferases, hydrolases, lyases, isomerases, and ligases [11] following Zn deficiency [10,11,18]. On the other hand, an increase was also observed in 100 and 500 µM Cd treatments compared to the control along with the overexpression of the GR and GST genes. GR and GST show similar patterns; however, in these cases, there was also a statistically proven overexpression compared to the control. This phenomenon indicates that Cd stress was severe enough to activate both enzymatic and non-enzymatic antioxidant systems in Cd-stressed maize seedlings [40]. Mahmoud et al. [84] proved that Cd toxicity increased both the levels of non-enzymatic antioxidants, as indicated by higher FRAP values in their case, and the overexpression of AtFeSOD gene as well in maize lines with different Cd tolerance levels. However, for 100 µM Zn, GST expression levels were higher than those of the control, which in this case indicates the formation of deficiency symptoms that also activate antioxidant defense mechanisms since the function of GST is to couple xenobiotics to glutathione, resulting in less toxic forms to compensate for the metabolic imbalance and reduced growth [9] by deficiency-caused loss of enzyme function [10,11,18]. In a review work by Hänsch and Mendel [85], they summarized that Zn is essential for carbonic anhydrase, the limiting enzyme for CO_2_ fixation in C4 plants, for Cu-Zn superoxide dismutase, and D-ribulose-5-phosphate 3-epimerase [85,86] and the lack of this essential element may cause serious functional disorders in photosynthesis [86], DNA transcription, RNA processing, and translation [87]. Therefore, to overcome deficiency symptoms, an increased rate of antioxidative enzyme activity was found by Tewari et al. [88], which was also reflected in the increased rate of GST not only for high (toxic) concentrations but also in the case of deficiency. Heavy metals inhibit GST function or reduce glutathione (GSH) concentrations, which would protect plants by altering the GSH/GSSG balance [89,90,91]. Moons [92] found that Cd, Co, Ni, and Zn enhance the expression of GST genes osgstu3 and osgstu4, indicating that GST is associated with metal detoxification. Our results further strengthen this role of heavy metals in GST function and inhibition.

### 3.5. Summary and Future Perspectives of This Research

Based on the evaluation of the parameters and the outcome of the PCA analysis, the different physiological effects of Zn and Cd were separated, as were the effects of the different (essential-toxic) concentrations of Zn. It can be stated that the lower Zn (100 µM) was in the range of Zn deficiency with positive effects being recorded specifically at the concentration of 1000 µM, when higher initial values for DF suggest better overall plant condition, whereas 2000 µM Zn concentration resulted in toxic symptoms similarly to the effect of the nonessential Cd. We found that the physiological reactions, associated with antioxidants and lipid oxidation, were evident in the changes in non-invasive biophoton emissions, specifically in the decrease in DF [89] and the resulting variations in UWLE intensities, as described in previous works as well [34,42,76].

As a continuation of this research, the function of Zn-dependent enzymes would be worthwhile to investigate in order to obtain a more precise picture of the essential-toxic transitions, such as the ubiquitous CuZn-SOD enzymes [93], localized in the cytoplasm, the function of which is directly related to the endogenous Zn level in plants [88]. This would also allow the Zn concentrations used in the present work to be extended to additional intermediate concentrations between 1000 and 2000 µM, thus providing a more accurate assessment of the specific functional transitions.

## 4. Materials and Methods

### 4.1. Growing Conditions

**Figure 11:**
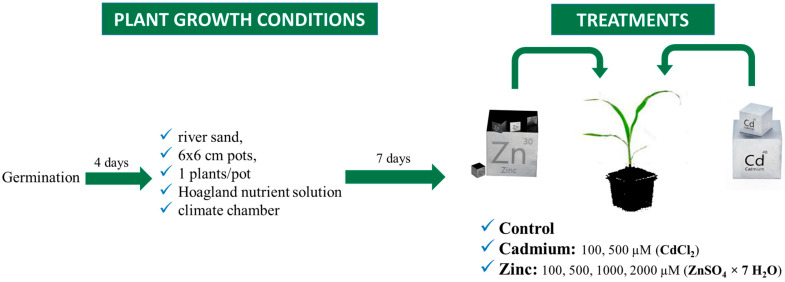
Growing and treatment procedures and parameters.

Maize seeds were germinated in filter paper for 4 days. The seeds were placed on the filter paper, rolled up and soaked in distilled water. The germinated seeds were planted in 6 × 6 cm plastic pots filled with river sand. The plants were placed in a Pol-Eco Apartura KK 1450 climate chamber (POLEKO-APARATURA sp.j. ul. Kokoszycka172C 44–300 Wodzisław Śląski, Poland) at 20 °C, 700 µM m^−2^ s^−1^ light intensity for 16 h as daylight conditions and 16 °C; 0 µM m^−2^ s^−1^ light intensity for 8 h as night conditions and grown for one week, watered with Hoagland solution (Figure 11). During the growing period, 56 plants (plants/treatment) were selected for the measurements.

### 4.2. Treatments

For each treatment, 8 plants were used for the measurements. The heavy metal treatments were applied with zinc sulfate (ZnSO_4_ × 7 H_2_O) solution at concentrations of 100 µM, 500 µM, 1000 µM and 2000 µM for Zn and Cd (CdCl_2_) at concentrations of 100 µM and 500 µM (Figure 11), simultaneously with bi-daily watering, applied in 10–10 mL volumes, for Zn and Cd treatments in addition to the control group with a 4 × dilution of Hoagland nutrient solution for water and nutrient supply for the control and Cd-treated plants, and for the Zn-treated plants, the supplementation of the nutrient solution with Zn was achieved by the appropriate concentrations. The treatments lasted for 6 days, after which the non-invasive measurements and the sampling for analytical measurements took place.

### 4.3. Measurement of Physiological Parameters

**Figure 12:**
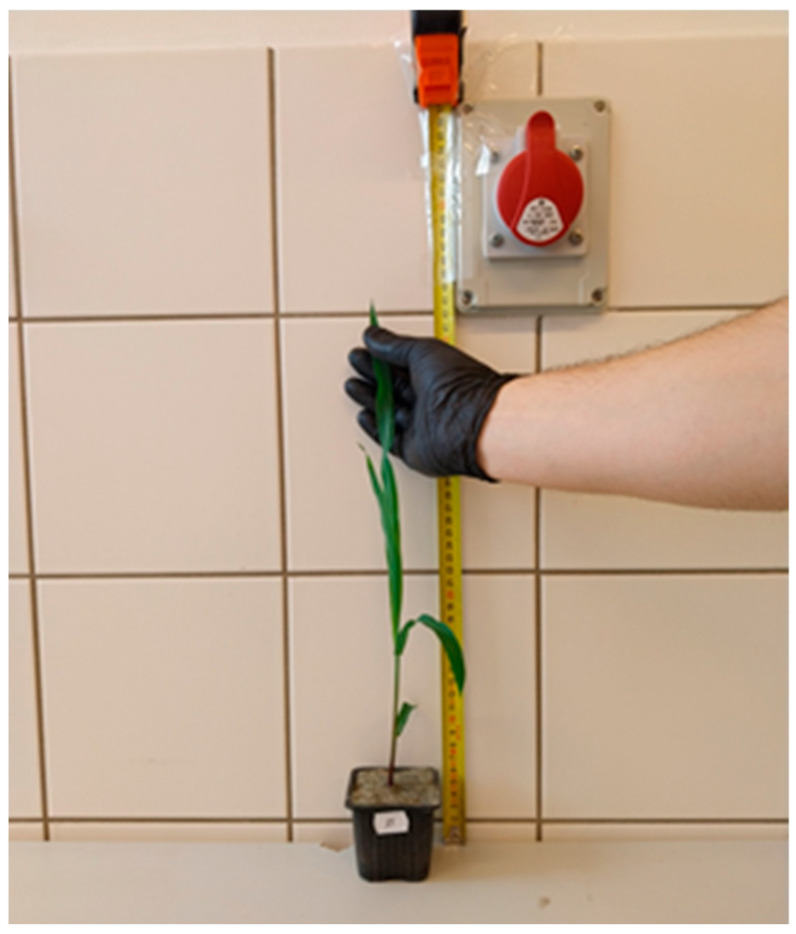
Plant length measurement.

The height of the plants was measured on the 6th day after the treatments using a tape measure (Figure 12). In all cases, the height of the growing pot (6.5 cm) was subtracted from the height measurements. Also, on the same day, the weight of the plants was measured on an analytical balance individually (OHAUS Discovery DV215CDM (OHAUS CORPORATION, 1.800.672.7722, 8 Campus Drive, Suite 105, Parsippany, NJ 07054, USA). Leaves were cut and mixed thoroughly and from this average sample, 0.1–0.1 g per treatment were placed in aluminum foil for analytical measurements and 0.03 g for gene expression assays. After sampling, plant samples were immediately stored at −20 °C and samples for gene expression assays at −80 °C.

### 4.4. Non-Invasive Measurements

#### 4.4.1. Estimation of Relative Chlorophyll Content

**Figure 13:**
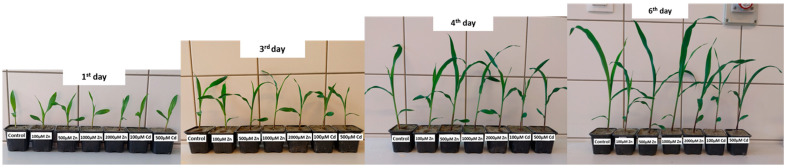
Effect of different heavy metal treatments (100, 500, 1000, 2000 µM Zn and 100 and 500 µM Cd) on the growth of maize seedlings.

The relative chlorophyll content of plants was measured with a Minolta SPAD 502 (Konica Minolta, Europaallee, 17 30855 Langenhagen, Germany), Figure 13. It can measure the intact transmission of plants at wavelengths between 650 and 940 nm. The chlorophyll content and the SPAD index value are closely correlated and therefore the result of the measurement provides an estimate of the chlorophyll content. Measurements are made by briefly enclosing the leaf inside the sensor, which is 2 × 3 mm. Three plants per treatments were measured, 10 values for each plant were collected from different parts of the leaf surface.

#### 4.4.2. Measurement of the Normalized Difference Vegetation Index

The Normalized Difference Vegetation Index (NDVI) is used to characterize the intensity of the green color of a plant, as the chlorophyll pigment of a healthy plant absorbs most of the visible red light, while the cell structure of the plant reflects most of the near infrared light. The instrument used for the measurement was a PolyPen RP 410 (PSI (Photon Systems Instruments) spol. s r.o., Průmyslová 470, 664 24 Drásov, Czech Republic), which, when clipped onto the measuring surface of the leaf, measures reflectance and the determination of vegetation index values: the selected leaf is placed under the leaf clip and the measurement is conducted with the press of a button in a similar way as described for SPAD index measurement (Section 4.4.1).

#### 4.4.3. Fluorescence Induction Measurement

Fluorescence induction measurements can be used to investigate the physiological state of plants through the photosynthetic activity of the photosystem II (PSII). The instrument used was a FluorPen FP 110/D (PSI (Photon Systems Instruments) spol. s r.o., Průmyslová 470, 664 24 Drásov, Czech Republic). Leaves were dark-adapted for 30 min via sealed clips being placed on them. When the device was inserted into the staple, the seal was removed and F_v_/F_m_ values (maximum quantum efficiency of the PSII) were determined.

#### 4.4.4. Biophoton Emission Measurement

Detection of biophoton emission was performed using a NightSHADE LB 985 In Vivo Plant Imaging System (Instutute Berthold Technologies Bioanalytical Instruments, Calmbacher Strasse 22, D-75323 Bad Wildbad, Germany), which featured a a sensitive, thermoelectrically cooled slow-scan NighOwlcam CCD device cooled to −68 °C. The exposure time was 60 s using a pixel binning of 4 × 4 and both the “background correction” and the “cosmic suppression” options were enabled to ensure the elimination of high-intensity pixels potentially caused by cosmic radiation. In order to obtain a standardized starting value of DF, prior to quantifying biophoton emission, LED panels with maximum intensities of far red (730 nm), red (660 nm), green (565 nm), and blue (470 nm) light were applied for a duration of 5 s. Then, the LEDs were deactivated and luminescence was observed for several minutes, the photon counts were gathered in every 60 s and analyzed using IndiGO™ 2.0.5.0. software. The resulting counts per second (cps) values were then converted to counts per second per square millimeter (cps/mm^2^) and adjusted for area. This form of biophoton emission suggests a stress state at a lower emission signal, with the photosynthetic apparatus of these plants being disturbed or stressed.

UWLE was also measured with this instrument. After the final DF measurements, one detached leaf per treatment was placed in the instrument and isolated from the light source. The 5 s illumination was followed by a 60 min measurement period in the dark. In this case, the first 30 min are not considered, as the DF phenomenon disappears in about this time, followed by UWLE. The latter is not a photosynthetic-related photon emission, but originates from oxidative metabolic processes in plants and increases UWLE values.

Images taken during the measurements were saved by IndiGO™ 2.0.5.0. software and the biophoton emission rate was expressed in counts per second (cps). The cps from selected areas in the images were divided into units of millimeters to give the number of photons emitted per millimeter by a plant.

### 4.5. Analytical Measurements

#### 4.5.1. Measurement of Zn and Cd Content

The element analysis was carried out according to the standardized methods (MSZ-08-1783-33:1985, MSZ-08-1783-17:1984 5.1) of the Central Laboratory of the Hungarian University of Agriculture and Life Sciences, Hungary. The measurements were carried out with the following instrumentation: Ohaus Adventurer Pro analytical scale (OHAUS CORPORATION, 1.800.672.7722, 8 Campus Drive, Suite 105, Parsippany, NJ 07054, USA), Nabertherm LT24/11/P330 muffle furnace (Nabertherm GmbH, Bahnhofstr. 20, 28865 Lilienthal, Germany), Memmert WNB 22 water bath (Memmert GmbH + Co., KG Äußere Rittersbacher Straße 38 91126 Schwabach, Germany) and JY Ultima2 ICP-OES (HORIBA Advanced Techno, Co., Ltd. 31, Miyanonishi-cho, Kisshoin Minami-ku Kyoto 601-8306, Japan).

#### 4.5.2. Measurement of Ferric Reducing Antioxidant Power

Ferric reducing antioxidant power (FRAP) measurement was performed using the method of Benzie and Strain (1999) [51]. FRAP reagent consists of 300 mM sodium-acetate buffer (pH 3.6), 10 mM TPTZ (2, 4, 6-tripyridyl-s-triazine) in 40 mM HCl and 20 mM FeCl_3_ × 6H_2_O of which the working FRAP reagent was prepared as follows right before the measurement: acetate buffer, TPTZ and FeCl_3_ × 6H_2_O in the ratio of 10:1:1 along with 1000 µM ascorbic acid as the standard solution.

Plant samples of 0.1 g were ground in cooled mortar in 1.5 mL of phosphate buffer (pH 7.6) and quartz sand, then placed in 2 mL Eppendorf tubes and centrifuged (Hettich, MIKRO 220R; Andreas Hettich GmbH & Co., KG Föhren str. 12, Tuttlingen, Germany) for 10 min at 4 °C at 13.000 rpm. Following centrifugation, 1950 µL reagent and 50 µL sample was added to 2 mL Eppendorf tubes and incubated immediately at 37 °C for 15 min. The absorbance was measured by BIORAD SmartSpec^TM^ Plus spectrophotometer (Bio-Rad Ltd., 1000 Alfred Nobel Drive Hercules, CA, USA) at 593 nm and the values are given in µg AS equivalent g^−1^ fresh weight.

#### 4.5.3. Lipid Oxidation Measurement Based on Malondialdehyde (MDA) Determination

Lipid oxidation was determined by the measurement of malondialdehyde (MDA) method developed by Heath and Packer (1968) [94] with some modifications.

Plant samples of 0.1 g were ground in 1.5 mL of 0.1% trichloroacetic acid (TCA) solution in a freezer-cooled rubbing mortar and placed in 2 mL Eppendorf tubes and centrifuged for 10 min, 4 °C, 13.000 rpm in a Hettich, MIKRO 220R centrifuge (Andreas Hettich GmbH & Co., KG Föhren str. 12, Tuttlingen, Germany). A sample of 0.375 µL of the extracts was then transferred to a 5 mL screw cap tube containing 1125 µL of reagent (20% trichloroacetic acid (TCA), 0.5% thiobarbituric acid (TBA)) and incubated for 30 min at 96 °C. After that, the samples were cooled down to room temperature before measuring the absorbance with a BIORAD SmartSpecTM Plus spectrophotometer (Bio-Rad Ltd., 1000 Alfred Nobel Drive Hercules, CA, USA) and MDA concentration was calculated by subtracting the non-specific absorption at 600 nm from the absorption at 532 nm using an absorbance coefficient of extinction, 156 mM^−1^cm^−1^. The results were expressed as nM g^−1^ fresh weight.

### 4.6. Gene Expression Studies

Thirty mg each of leaf samples were homogenized in lysis buffer of RNeasy Tissue Mini Kit (Qiagen, 19300 Germantown Road, Germantown, MD, USA) in a TissueLyser II high-throughput sample homogenizer (Qiagen, 19300 Germantown Road, Germantown, MD, USA). To avoid RNA degradation, the homogenizer adapter was cooled to −20 °C. RNA extraction was performed according to the manufacturer’s instructions. RNA quantity and quality were measured using a Thermo Scientific™ NanoDrop™ OneC Microvolume UV-Vis spectrophotometer (Thermo Scientific™ 840274200, 168 3rd Ave, Waltham, MA, USA). Synthesis of cDNA was performed using the QuantiTect Reverse Transcription Kit at 42 °C for 15 min (Qiagen, 19300 Germantown Road, Germantown, MD, USA) according to the manufacturer’s instructions, after DNAase digestion for 2 min at 42 °C. Following cDNA synthesis, PCR reaction was performed with the primers listed in Table 2.

The reaction conditions were applied as follows: initial denaturation at 95 °C for 15 min; 40 PCR cycles of 30 s each at 95 °C, 60 s at 60 °C and 1 min at 72 °C. The amplicons were identified by melting point analysis and relative gene expression levels were determined from the threshold cycle values using the 2^−ΔΔCT^ method of Livak and Schmittgen (2001) [96]. In addition, melting point analysis of the amplicons per primer pair was performed to confirm amplification of a given gene product. This method also allows comparisons of gene expression values between genes and treatments.

### 4.7. Statistical Analysis

The experimental data were tabulated using Microsoft Excel software (v.16.0), means and standard deviations were calculated, and the results were evaluated by one-way ANOVA (*p* < 0.05) and Duncan’s post hoc test using IBM SPSS 20.0 statistical software.

During the initial preparation of the data for PCA, the min-max normalization method was used to achieve order-of-magnitude comparability of the values. This method describes all characteristics in the same range (0–1), ensuring objectivity and precision in the evaluation process. As a next step, the principal component analysis (PCA) was applied to identify those variables which influenced the variations in the dataset at the highest level. The results and plots of the PCA were calculated and drawn by the “devtools” [97] and “ggplot2” [98] packages in R (version 4.3.3). As a next step, the strength of the relationship between the variables was determined using the Pearson correlation. The Pearson correlation coefficients were illustrated by heat maps using R.

## 5. Conclusions

The current research investigated how different levels of Zn affect plant reactions, specifically focusing on photosynthetic and oxidative metabolic activities. The experiment intended to differentiate the positive and negative impacts of Zn on plants in order to separate the essential and toxic functions of this important micronutrient in maize production.

The lower Zn concentration (100 µM) indicated deficiency, while 1000 µM showed positive effects on the functionality of the maize plants. The dosage of 2000 Zn µM caused toxic symptoms similar to Cd in antioxidants and lipid oxidation, in the decrease in DF and the resulting variations in UWLE intensities. To better understand the essential–toxic transitions, it is important to study the function of the Zn-dependent enzymes, such as CuZn-SOD enzymes, which are ubiquitous in the cytoplasm and directly related to plant Zn levels. The Zn concentrations used in this study could be extended to intermediate levels between 1000 and 2000 µM to enable a more accurate assessment of the functional transitions. The selection of the appropriate concentration of Zn supplementation will contribute to the development of a more sustainable and at the same time more cost-effective micronutrient supplementation technology for more successful maize production.

## Figures and Tables

**Table 1:** Changes (higher values: +; lower values: −; no change: =) in each parameter compared to the control in the given treatments and measurements.

Measurement	100 µM Zn	500 µM Zn	1000 µM Zn	2000 µM Zn	100 µM Cd	500 µM Cd
Shoot height	−	=	+	−	+	−
Shoot weight	−	+	+	−	+	−
SPAD	−	−	−	−	−	−
NDVI	−	−	−	−	−	−
F_v_/F_m_	−	+	+	−	−	−
DF	−	+	+	−	−	−
UWLE	+	+	+	+	+	+
MDA	+	−	+	+	+	+
FRAP	−	−	+	−	+	+
GST	+	+	−	+	+	+
GR	−	+	−	+	+	+
LOX	+	+	−	+	+	+

**Table 2:** Primers used for gene expression studies.

Name	Sequence	Adhesion Temperature (°C)	Reference
MEP	F: TTCCTCATGTTCTTCGTGCC/ R: CAGTTCTCATTCCATCCGTG	61.3	Manoli (2012) [95]
GST	F: GACCATCAACTTCGCCACC/ R: ATCTACAAGTCACCATCCTGC	62.7	Oláh (2020) [50]
GR	F: GGAACCTACCAAACCAGATTA/ R: GGCAACGAAGACATCAACATC	60.4	Oláh (2020) [50]
LOX	F: CCCAACAGCATTTCCATCTG/ R: CCAATTACCACGCTTCTCATTC	62.3	Oláh (2020) [50]

## Data Availability

The raw data supporting the conclusions of this article will be made available by the authors on request.
